# Sick Building Syndrome among Junior High School Students in Japan in Relation to the Home and School Environment

**DOI:** 10.5539/gjhs.v8n2p165

**Published:** 2015-06-11

**Authors:** Motoko Takaoka, Kyoko Suzuki, Dan Norbäck

**Affiliations:** 1 Department of Biosphere Sciences, School of Human Sciences, Kobe College, 4-1 Okadayama, Nishinomiya Hyogo, Japan; 2 Department of Medical Sciences, Occupational and Environmental Medicine, Uppsala University and University Hospital, Uppsala, Sweden

**Keywords:** Sick building syndrome (SBS), Japan, home environment, school environment, allergy

## Abstract

**Purpose:**

There is an increasing concern about sick building syndrome (SBS), especially in Asia. The aim of this study is to investigate associations between SBS and the home, school environment and personal factors among Japanese junior high school students.

**Methods:**

We investigated students in four junior high schools in Hyogo in Kansai area, Japan. A questionnaire study was performed among students (n=1056), 12-15 years old. Temperature and relative air humidity was measured in the classrooms and dust was collected from the classroom floors and air and was analysed for cat and dog allergens. Associations were analysed by multi-level logistic regression.

**Results:**

Mucosal symptoms (45.4%), general symptoms (38.9%) and skin symptoms (22.6%) were common. Totally 8.8% reported cat allergy, 6.1% dog allergy, 6.0% mold allergy and 25.7% pollen allergy. Atopy, window pane condensation, floor dampness and odor at home and high relative air humidity in the classrooms were associated with SBS.

**Conclusion:**

The prevalence of SBS symptoms was high and associated with both home and school environment. Window pane condensation and floor dampness at home can increase the risk for SBS symptoms in students. Moreover high relative air humidity at school may increase the risk for SBS.

## 1. Introduction

Sick building syndrome (SBS) was initially defined empirically since in certain office buildings, a large proportion of subjects reported similar symptoms that they attributed to the indoor environment (WHO, 1982). The term SBS has been used to describe symptoms (including headache, fatigue and irritation in upper respiratory tract, nose throat, eyes, hand and or facial skin) that can be influenced by the indoor environment (Redlich et al., 1997; WHO, 1983).

SBS symptoms are related to both personal and environmental risk factors. Previous studies have shown that personal vulnerability and psychosocial factors are associated with SBS symptoms, including female gender and history of allergic disorders (Norback et al., 1990; Niven et al., 2000) as well as psychosocial work stress (Bakke et al., 2007; Runeson & Norback, 2012). Many cross-sectional epidemiological and some experimental studies have indicated that indoor exposure factors such as building dampness, bioaerosols, volatile organic compounds (e.g. formaldehyde, toluene), indoor mold, house dust mite allergens and certain building characteristics (e.g. low ventilation rate) may be associated with SBS symptoms (Apter et al., 1994; Sundell et al., 2011; Saijo et al., 2011). However the mechanism causing the symptoms is still not known.

There is increasing concern about these types of symptoms, especially in Asia (Norback, 2009; Zhang et al., 2011a) and especially in Japan (Imai et al., 2008; Nakayama & Morimoto, 2009). In Japan, the main focus has been on the home environment. From the 1990s, the number of people suffering from sick house syndrome (SHS) in Japan has increased. SHS closely resembles SBS. SBS is mainly reported in offices and sometimes schools while SHS is found in dwellings. One Japanese study identified dampness as a major cause of SHS in public apartment houses (Saijo et al., 2009). Other studies on SHS in new dwellings in Japan demonstrated that there are associations between SHS and dampness, formaldehyde, alpha-pinene (Takeda et al., 2009) as well as elevated indoor levels of aldehydes and aliphatic hydrocarbons (Takigawa et al., 2012).

Data from of six areas in Japan indicated that there are associations between SHS and different types of odors and water leakage at home (Nakayama & Morimoto, 2009).

For school children, beside the home environment, the school environment is an important indoor environment. Studies from Western countries have shown that the school environment may exacerbate asthma symptoms, allergic reactions and other respiratory symptoms (Daisey et al., 2003). Furry pet allergens are transferred to the school from homes by contamination of clothes (Salo et al., 2009). SBS may have important physical and educational implications for school children. Dampness and mold have been reported to be common in schools in Europe (Haverinen-Shaughnessy et al., 2012) and associations between dampness in schools and SBS symptoms in school children have been reported from Sweden (Ahman et al., 2000) and Japan (Saijo et al., 2010). One Chinese study demonstrated that temperature, relative air humidity, NO_2_ and SO_2_ in classrooms were associated with various SBS symptoms in school children (Zhang et al., 2011a).

Although many reports on SHS have been published from Japan, there are few studies on SBS symptoms in Japanese school children. Since children are exposed to different indoor environments, it is an advantage to include both school and home environment in the same study. We found only one study which did that, a Japanese study on associations between school dampness and children’s health (Saijo et al., 2010). The first aim of our study is to investigate associations between the prevalence of SBS symptoms among school children and selected factors in the home environment. The second aim is to study associations between SBS symptoms and indoor climate and allergen levels in the school environment. Finally, we investigated associations between SBS symptoms and selected personal factors (age, sex and atopy).

## 2. Materials and Methods

### 2.1 Study Population

We investigated students in four junior high schools in Hyogo in Kansai area, Japan, in 2008 and 2009 (in early summer both years). We selected the schools in an arbitrary way, including schools that were willing to participate. None of these four schools had any previous history of indoor environmental problems or health complaints reported to the school authorities. One of the schools was a public school, three were private schools and among the private schools, two were only for girl students.

Students in the public school and two of the private schools had school uniforms, which were only used in the school. All three grades were included in the study. Totally 32 classrooms in the four schools were randomly selected (6 classrooms in school A,4 classrooms in school B, 6 classrooms in school C and 16 classrooms in school D) and all students in these classrooms (n=1056) received a questionnaire in Japanese. The questionnaires were distributed in the school by the class teachers and answered by the students in the classroom. The students were 12-15 years old. The students used the same classrooms for most classes except for music, art and sport. The questionnaire survey was carried out one week before the classroom measurements. All personal information from questionnaire was kept confidential. The study was approved by the ethical committee of Kobe college and all participants gave informed consent.

### 2.2 Assessment of Demographic Data and Health Data

The self-administrated questionnaire included questions about symptoms compatible with SBS, based on previous SBS studies in Sweden (Sahlberg et al., 2012; Runeson-Broberg and Norback 2012) and in Chinese schools (Zhang et al., 2011a,b). The symptoms included; facial and hand rash or itching; eczema; eye irritation; swollen eyelids; nasal catarrh and obstruction; dryness in the throat; sore throat; irritating cough; headache; nausea; sensation of getting a cold; and tiredness. Each question had four alternative answers: ‘Yes, daily’; ‘Yes, 1-4 times/week’; ‘Yes, 1-3 times/month’; and ‘No, never’. The recall period was three months. Moreover, the questionnaire contained questions on age, sex and allergy to cat, dog, mold and pollen. The question on cat allergy asked: Do you have allergy to cats (yes, no, do not know): The question on dog allergy asked: Do you have allergy to dogs (yes, no, do not know): The question on cat allergy asked: Do you have allergy to mold (yes, no, do not know): The question on pollen allergy asked: Do you have allergy to pollen (yes, no, do not know). Not know and no was coded 0 and yes was coded 1 in the statistical analysis. There were no questions on body mass index (BMI) or the parents’ socio-economic status or occupation.

### 2.3 Assessment of Exposure

The questionnaire contained questions on exposure indicators in the current home environment, including type of dwelling, construction materials of the house, pet keeping (including type of pets) and environmental tobacco smoking at home (yes/no). Moreover there was one question on recent indoor painting (Wieslander et al., 1997) and one on new floor materials introduced during the last 12 months. There were four questions on current building dampness or mold growth in the last 12 months (Norback et al., 1999; Gunnbjornsdottir et al., 2006) and one additional question on other types of odor than moldy odor. The question on floor dampness asked about bubbles or yellow discoloration on plastic floor covering or black discoloration on wood floor. Finally, there was one question on window pane condensation in winter time, an indicator of both low air exchange and high relative air humidity.

### 2.4 Climate Measurements

Room temperature and relative air humidity were measured by a direct reading instrument (As one, TH-321) in empty classrooms in the weekend during one hour because the school did not permit any measurements during weekdays. In addition, temperature and relative air humidity were measured outside the school. At the same time in the weekend, vacuumed dust was collected in the classrooms by vacuum cleaning on special filters. After vacuum cleaning, two pairs of Petri dishes were placed in each classroom. They were kept open for seven days in each classroom and were closed and collected the next weekend.

### 2.5 Allergen Measurement

Two samples of settled dust were collected by vacuum cleaning in each room by dividing the room into two parts, one entrance-side half and one window-side half (Kim et al., 2005a; Zhao et al., 2006). The dust was collected from floors with a vacuum cleaner (300W) provided with a special dust collector (ALK Abello, Copenhagen, Denmark) equipped with a Millipore filter (pore size 6 μm). Vacuum cleaning of the floors was performed for 2 minutes per sample as in previous school studies (e.g. Smedje et al., 1997). The filters were sealed with a lid and stored in plastic bags at -20°C until extraction. Airborne settled dust was collected using two pair of Petri dishes in each classroom with base and lid facing upward on horizontal surfaces on corridor side, placed on top of bookshelves or similar area (1.8-2.2 meters above the floor) and kept open for seven days (Karlsson et al., 2002). The Petri dishes were closed and stored at room temperature until extraction. Petri dish samples and vacuumed dust samples were extracted as in previous studies (e.g. Zhao et al., 2006). Allergen levels were determined using two-site sandwich ELISA for cat (Fel d 1), dog (Can f 1) (Indoor Biotechnologies Ltd, Manchester, UK), as previously described (Kim et al., 2007). The allergen concentration in vacuumed dust was expressed per gram dust while the allergen contamination in the Petri dish samples was expressed per surface area (m2) and day.

### 2.6 Statistical Methods

Differences between groups were analyzed by Chi^2^ test and Mann-Whitney U test. Initially, associations between the health variables and indoor environmental factors were analyzed by multiple logistic regression analysis adjusting for age, sex and atopy. One model included 12 home environment factors, and another model included seven exposures in the school. Exposure at school was addressed to the students on classroom level. Both of models used mutual adjustment. Because of the hierarchic structure of the data, analysis of school data was performed by multilevel logistic regression, using 3 levels (student, classroom, school). Finally, all independent variables from the previous models were entered and non-significant factors were removed through backward stepwise regression (Wald) using p<0.1 as inclusion criteria. The associations were expressed as odds ratios (OR) with a 95% confidence interval (CI). Statistical analysis were performed with the Statistical Package for the Social Sciences (Stata Crop LP, College Station, Texas, USA SPSS Inc., Chicago, Illinois, USA) 19.0. Multilevel mixed logistic regression analysis was performed by STATA version 11.0. A 5% level of significance was applied in all analysis.

## 3. Results

The participation rate of the students was 99.2%, totally of 1048 students participated from the 32 classes. Totally 64.1% were girls and the average age was 13.4 years old (standard deviation (SD) =0.9, range 12-15 years old).

### 3.1 Questionnaire Data

**Figure 1:**
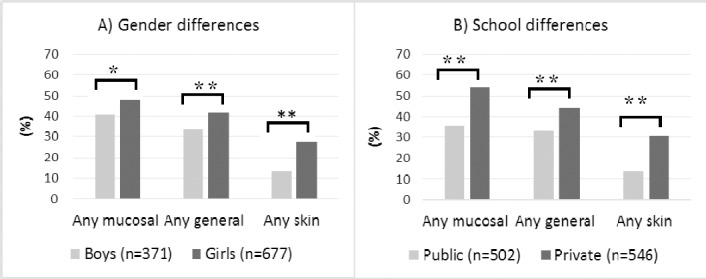
Gender and school type differences of three months prevalence of symptoms (n=1048).

Totally 13.4% had doctors’ diagnosed asthma, 8.8% cat allergy, 6.1% dog allergy, 6.0% mold allergy and 25.7% reported pollen allergy. About one third had any type of allergy (atopy).The most common types of symptoms were tiredness, nasal catarrh and sore throat while swollen eyelids and nausea were least common (Table 1). The prevalence of any weekly mucosal, general or skin symptoms was high. All types of SBS symptoms were more common in female students and in students from private schools (Figure 1).

**Table 1:** The prevalence of allergies and type of symptoms among students (n=1048)

	(%)
Cat allergy	8.8
Dog allergy	6.1
Mold allergy	6.0
Pollen allergy	25.7
Atopy (cat, dog, mold, pollen allergy)	32.3
Symptoms last 3 months	
Eye irritation	15.9
Swollen eyelids	3.3
Nasal catarrh	26.3
Nasal obstruction	24.2
Dryness in throat	10.9
Sore throat	24.0
Irritative cough	10.5
Any mucosal symptoms (7 symptoms)^a^	45.4
Headache	12.8
Tiredness	34.6
Sensation of getting a colld	4.6
Nausea	3.6
Any general symptoms(4 symptoms)^b^	38.9
Facial itching	6.2
Facial rash	13.2
Itching on the hands	9.2
Rashes on the hands	4.0
Eczema	5.9
Any skin symptoms (5 symptoms)^c^	22.6

a : At least one weekly symptom classfied as mucosal symptoms (eye irritation, swollen eyelids, nasal catarrh, nasal obstruction, dryness in the throat, sore throat, and irritative cough);

b : At least one weekly symptom classfied as general symptoms (headache, Tiredness, sensation of getting a cold and nausea);

c : At least one weekly symptom classfied as skin symptoms (facial itching, facial rash, itching on the hand, rashes on the hand and eczema).

A) Gender differences, B) school differences

**Figure 2:**
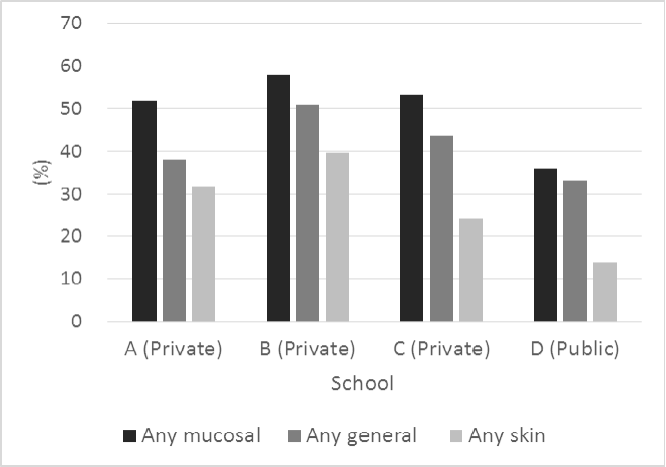
School differences of three months prevalence of symptoms (n=1048)

Any mucosal: At least one weekly symptom classfied as mucosal symptoms;

Any general: At least one weekly symptom classfied as general symptoms;

Any skin: At least one weekly symptom classfied as skin symptoms;

*:p<0.05, **:p<0.001;

Table 2 shows the prevalence of indoor factors in the dwellings. More than half of the students lived in a single family house. The status of home dampness was as follows: 8.6% reported visible mold, 1.3% moldy odor, 7.5% water leakage and 38.2% had window pane condensation in winter. Keeping a dog was more common than the keeping a cat. Exposure to environmental tobacco smoke (ETS) at home was common. When comparing the prevalence of SBS symptoms between schools, the private school B had most symptoms and the public school D had least symptoms (Figure 2).

**Table 2:** Indoor environmental factors in the dwelling

	(%)
Single family house	59.1
Wooden house	29.4

Indoor painting in the last 12 months	7.8
New floor in the last 12 months	4.6

Any pet keeping at home	36.1
Cat	4.2
Dog	19.9

Window pane condensation in winter	38.2
Indoor smoking (ETS)	29.4
Other odor than moldy odor	10.0

Any building dampness and mold in the last 12 months	
Water leakage	7.5
Signs of floor dampness	4.5
Visible mold	8.6
Moldy odor	1.3

Any mucosal: At least one weekly symptom classfied as mucosal symptoms;

Any general: At least one weekly symptom classfied as general symptoms;

Any skin: At least one weekly symptom classfied as skin symptoms.

### 3.2 School Environment

The outdoor temperature was 22.5-27.8 °C and the outdoor relative air humidity was 41-68%. Environmental data for the classrooms are given in Table 3. Cat and dog allergens were found in all settle dust samples. In the Petri dish air samples, most samples contained cat (96.9%) and dog (93.7%) allergens. There were no significant difference in levels of cat and dog allergens between private schools and the public school. The median levels of room temperature and indoor relative air humidity were higher in private schools and there were significant differences for temperature (Mann-Whitney U-test; p=0.001) and relative air humidity indoors (p=0.006).

**Table 3:** Indoor climate and allergen levels in settled dust and air in 32 classrooms

	N	Median	IQR	Min-Max
Temperature (°C)	32	24	23-25	22-27
Relative air humidity (%)	32	53.5	47.7-59.2	46.4-67.2
Classroom area (m^2^)	32	75.0	74.9-75.0	70.1-88.7
No. of students	32	33.5	29.3-35.0	17.0-47.0
Density (person/m^2^)	32	0.44	0.39-0.48	0.23-0.57
Fel d 1				
Floor dust (ng/g dust)	64^a^	41.0	23.4-92.4	2.1-409.5
Petri dish (air) (ng/m^2^/day)	64^a^	18.6	5.9-25.1	<1-67.6
Can f 1				
Floor dust (ng/g dust)	64^a^	101.1	53.9-101.1	7.1-535.7
Petri dish (air) (ng/m^2^/day)	64^a^	13.3	6.0-13.3	<1-243.0

a : Two samples/classroom.

In the multiple logistic regressions analysis, associations between SBS symptoms and personal factors as well as home environmental factors were found (Table 4). Having atopy was positively associated with mucosal symptoms, general symptoms and skin symptoms and skin symptoms were more common in female students. Among home environment factors, window pane condensation, signs of floor dampness and other odor than moldy odor were positively associated with mucosal symptoms, general symptoms and skin symptoms. Pet keeping was positively associated with mucosal symptoms only. Because of the strong correlation between relative air humidity and type of school, we had to use two different school environment regression models. In the first model, including age, sex, atopy and temperature, relative air humidity, student density in the classroom (persons/m^2^ floor area) and allergens, atopy was still associated with all types of SBS symptoms, and females had more skin symptoms. Among school environment factors, indoor relative air humidity was associated with mucosal and skin symptoms (Table 5). In the second model including age, sex. atopy, student density, type of school and allergens there were associations between attending a private school and mucosal (OR=2.22, 95% CI=1.58-3.13), general (OR=1.40, 95% CI=0.99-1.97) and skin (OR=2.41, 95% CI=1.60-3.62) symptoms (data not shown in the table). Finally, we analyzed associations including both personal factors, home and school environment factors in reduced multiple models obtained by backward stepwise regression (Table 6). Atopy, windowpane condensation, signs in the floor dampness, odor at home (other than moldy odor) and high relative air humidity at school were associated with mucosal symptoms, general symptoms and skin symptoms. Moreover, pet keeping at home was associated with mucosal symptoms and female gender was related to skin symptoms.

**Table 4:** Associations between weekly symptoms, personal factors and home environmental factors (mutual adjustment)

	Mucusal^a^	General^b^	Skin^c^
Female gender	1.26(0.97-1.66)	1.38(1.04-1.82)*	2.49(1.69-3.44)**
Age	0.89(0.77-1.02)	0.96(0.83-1.11)	0.94(0.80-1.12)
Atopy^d^	2.17(1.65-2.85)**	1.70(1.29-2.24)**	1.96(1.44-2.68)**
Single family house	0.87(0.65-1.17)	0.83(0.61-1.12)	0.93(0.65-1.32)
Wooden house	1.25(0.91-1.70)	1.16(0.85-1.59)	1.08(0.75-1.56)
Indoor painting	1.36(0.84-2.21)	0.85(0.51-1.41)	1.30(0.75-2.25)
New floor materials	0.73(0.38-1.38)	0.62(0.31-1.22)	1.13(0.56-2.29)
Window pane condensation	1.89(1.44-2.48)**	2.14(1.63-2.81)**	1.60(1.17-2.19)**
Water leakage	0.67(0.41-1.12)	0.96(0.58-1.59)	1.17(0.67-2.03)
Floor dampness	1.90(1.03-3.51)*	2.77(1.49-5.14)**	2.04(1.11-3.84)*
Visible mold	0.75(0.46-1.23)	0.76(0.46-1.26)	1.06(0.61-1.83)
Mouldy odor	0.73(0.23-2.28)	1.18(0.38-3.66)	2.40(0.74-7.72)
Other odor	1.67(1.06-2.65)*	1.81(1.15-2.86)*	1.77(1.09-2.85)*
Pet keeping	1.39(1.05-1.83)*	1.28(0.97-1.69)	1.21(0.87-1.67)
Indoor smoking (ETS)	0.90(0.78-1.03)	0.91(0.79-1.05)	0.94(0.80-1.11)

* p<0.05,

** p<0.01.

a : At least one weekly symptom classfied as mucosal symptoms;

b : At least one weekly symptom classfied as general symptoms;

c c: At least one weekly symptom classfied as skin symptoms;

d : Including either of cat, dog, mold or pollen allergy;

Age, sex, atopy and home environmental factors in the model.

**Table 5:** Associations between weekly symptoms, personal factors and measured school environmental factors (mixed-effects multi levels logistic regression)

	Mucosal^a^	General^b^	Skin^c^
Female gender	1.00(0.75-1.34)	1.24(0.93-1.66)	1.81(1.24-2.63)**
Age per year	0.98(0.84-1.14)	1.02(0.87-1.18)	1.10(0.91-1.33)
Atopy^d^	2.20(1.68-2.88)**	1.81(1.38-2.36)**	2.01(1.48-2.74)**
Temperature	0.91(0.79-1.04)	0.93(0.81-1.06)	1.01(0.86-1.19)
Relative air humidity^e^	1.81(1.34-2.45)**	1.29(0.96-1.75)	2.18(1.52-3.12)**
Density^f^	0.95(0.76-1.18)	0.81(0.65-1.01)	1.13(0.88-1.47)
Cat allergen on floor^g^	0.88(0.74-1.05)	1.02(0.85-1.22)	0.88(0.71-1.08)
Cat allergen air^h^	1.17(0.46-2.97)	1.12(0.44-2.84)	0.86(0.29-2.55)
Dog allergen floor^g^	0.99(0.88-1.11)	1.02(0.91-1.15)	0.98(0.85-1.12)
Dog allergen air^h^	0.96(0.62-1.48)	1.14(0.74-1.77)	1.14(0.72-1.81)

** p<0.01;

Age, sex, atopy and school environmental factors in the model;

a : At least one weekly symptom classfied as mucosal symptoms;

b : At least one weekly symptom classfied as general symptoms;

c : At least one weekly symptom classfied as skin symptoms;

d : Including either of cat, dog, mold or pollen allergy;

e : Odds ratio (OR) expressed as change of coefficient per 10%;

f : Odds ratio (OR) expressed as change of coefficient per 0.1 person /m^2^;

g : Odds ratio (OR) expressed as change of coefficient per 100 ng/g dust;

h : Odds ratio (OR) expressed as change of coefficient per 100 ng/m2/day.

**Table 6:** Associations between weekly symptoms, personal factors, home environment factors and measured school environmental factors in reduced multiple models, by backward stepwise regression

		OR	(95% CI)	p-value
Mucosal^a^	Female gender	1.81	(1.26-2.62)	0.002
	Atopy^d^	1.84	(1.34-2.52)	<0.001
	Window pane condensation at home	1.65	(1.21-2.24)	0.002
	Other odor at home	2.09	(1.15-3.80)	0.02
	Pet keeping at home	1.79	(1.11-2.89)	0.02
	Relative air humidity at school^e^	1.88	(1.47-2.41)	<0.001
General^b^	Atopy^d^	1.64	(1.24-2.17)	<0.001
	New floor	0.57	(0.30-1.11)	0.100
	Window pane condensation at home	2.11	(1.62-2.76)	<0.001
	Floor dampness at home	2.43	(1.35-4.38)	0.003
	Other odor at home	1.76	(1.12-2.76)	0.01
	Relative air humidity at school^e^	1.48	(1.21-1.82)	<0.001
	Density^f^	0.84	(0.69-1.02)	0.080
Skin^c^	Female gender	1.81	(1.26-2.62)	0.002
	Atopy^d^	1.84	(1.34-2.52)	<0.001
	Window pane condensation at home	1.65	(1.21-2.24)	0.002
	Floor dampness at home	2.09	(1.15-3.80)	0.02
	Other odor at home	1.79	(1.11-2.89)	0.02
	Relative air humidity at school^e^	1.88	(1.47-2.41)	<0.001

Age, sex, atopy, 12 home environmental factors and 7 school factors included.

a : At least one weekly symptom classfied as mucosal symptoms;

b : At least one weekly symptom classfied as general symptoms;

c : At least one weekly symptom classfied as skin symptoms;

d : Including either of cat or dog or mold or pollen allergy;

e : Odds ratio (OR) expressed as change of coefficient per 10%;

f : Odds ratio (OR) expressed as change of coefficient per 0.1 person /m^2^.

## 4. Discussion

In this study we found significant associations between both home environment factors and measured school environmental factors and SBS symptoms in Japanese students. In addition, personal factors such as gender, and atopy were associated with SBS symptoms.

There were significant associations between window pane condensation in winter, signs of floor dampness and other odor than moldy odor in the home and SBS symptoms. Moreover there were significant positive associations between relative air humidity in the classrooms and mucosal symptom and general symptoms. Private schools had more SBS symptoms which could be due to higher stress level. We found no previous epidemiological study investigating both home environment factors and measured school environment factors in relation to SBS symptoms among Japanese students.

The prevalence of mucosal symptoms, general symptoms and skin symptoms were relatively high. One previous Japanese study in elementary school students in Hokkaido reported lower levels of general symptoms (4.8%) and skin symptoms (11.3%) as compared to our results (Saijo et al., 2010). When comparing our results with other countries, one Chinese study in junior high students reported a similar prevalence of weekly mucosal symptoms (40.9%) and general symptoms (44.3%) as in our study but lower levels of skin symptoms (6.7%) (Zhang et al., 2011a). We found that females reported more skin symptoms. This is in agreement with many previous SBS studies reported a higher prevalence of symptoms in women as compared to men (Engvall et al., 2002; Saijo et al., 2009; Sahlberg et al., 2012).

In addition, we found associations between atopy (cat, dog, mold or pollen allergy) and SBS symptoms. This is in agreement with a previous Chinese school study (Zhang et al., 2011a) as well as previous SBS studies in adults (Sahlberg et al., 2012; Bjornsson et al., 1998). Atopy may affect to the personal sensitivity to factors causing SBS (Seki et al., 2007).

Japan is an island and Hyogo prefecture is near the coast. Because of this, outdoor relative air humidity is relatively high all the year around and results in high indoor air humidity levels in the buildings. We found associations between window pane condensation in winter and SBS symptoms. Window pane condensation occurs during the cold part of the year when there is poor ventilation in combination with high air humidity. One previous Japanese study found a positive association between window pane condensation and nose symptoms (Saijo et al., 2009). Moreover, Swedish studies have reported positive associations between window pane condensation and wheeze (Emenius et al., 2003) and exhaled NO, a biomarker of airway inflammation (Janson, 2005). Moreover, window pane condensation in winter has been shown to be associated with measured indoor exposures in homes, including increased relative air humidity and increased levels of house dust mite allergens in dust and poor ventilation flow will increase the levels of various indoor air pollutants (Emenius et al., 2000).

We found associations between signs of floor dampness and SBS symptoms which is in agreement with two previous studies (Wieslander et al., 1999; Bornehag et al., 2005). Floor dampness may occur due to capillary transportation of water from soil to the concrete slab, but also because of the design of the floor construction. A construction with thermal insulation between the soil and concrete slab is a safe and dry construction while thermal insulation between the concrete slab and house is a risk construction and commonly get dampness problem because the temperature profile cause high relative air humidity in the building materials (Cai et al., 2011). Our question on floor dampness asked about bubbles under PVC floor coverings or blackened wood floors. Bubbles under polyvinyl chloride (PVC) floors can occur when there is a degradation of the floor glue at elevated humidity levels in the concrete. Blackening of wood floors occur when tannin in the wood reacts with ammonia emitted from the damp floor construction. Emission of ammonia can occur from additives in concrete, and indoor problems due to ammonia emissions from concrete have been described from China and Japan (Lindgren, 2010). Moreover, elevated levels of ammonia have been measured in buildings with technically verified dampness in the floor construction (Wieslander et al., 1999). Building dampness in concrete floors may cause chemical degradation of building material, including degradation of phthalate esters in PVC or polyacrylate materials in floor coating or water-based floor glue, causing emission of 2-ethyl-1 hexanol in the air (Wieslander et al., 1999). Emission of 2-ethyl-1 hexanol can increase the prevalence of SBS symptoms (Wieslander et al., 2010; Ernstgard et al., 2010). One study reported that installation of ventilated floor in school with floor dampness resulted in positive health effects among personnel and pupils (Ahman et al., 2000).The prevalence of other signs of dampness such as visible mold, moldy odor or water leakage were lower than in the previous studies from Hokkaido, Japan (Saijo, 2009; Takeda et al., 2009) and not associated with SBS symptoms in our study.

Moreover, our study showed that other odor than moldy odor in home, was associated with all types of SBS symptoms. In previous studies, odor at home was reported to be associated with SBS symptoms (Nakayama & Morimoto, 2009; Saijo et al., 2011; Engvall et al., 2002). Perception of odor was related to both symptoms and reports on building dampness suggesting that the odor may be caused by hidden fungal growth. Indoor micro-organisms can produce specific microbial volatile organic compounds (MVOC), some which emit moldy or other odor. One Swedish study in day care centers reported there was a significant association between total fungal DNA in dust and any odor not perceived as moldy odor (Cai et al., 2009), suggesting that other odor than mold odor can be an indicator of mold contamination.

Cat and dog allergen contamination were common in our Japanese schools. This finding is in agreement with previous school studies from other countries (Kim et al., 2007; Zhao et al., 2006; Salo et al., 2009). Cat and dog allergens are transferred to the school environment from homes by contamination of clothes (Salo et al., 2009; Smedje & Norbäck 2001) and hair (Karlsson & Renström, 2005). However in our study, levels of cat and dog allergen in floor dust were much lower than reported from previous school studies from Sweden (Kim et al., 2005b; Smedje et al., 1997) or USA (Permaul et al., 2012). The high cleaing frequency in Japanese schools and the low prevalence of pet keepers could explain the low allergen levels. The levels of cat and dog allergens in the classroom were not associated with SBS symptoms in our study.

We found positive associations between relative air humidity in the classrooms and all types of SBS symptoms. In contrast, one study from Chinese schools found a negative association between relative air humidity in the classrooms and SBS symptoms, but they measured the air humidity during weekdays with full class (Zhang et al., 2011a). In our study, relative air humidity was measured in the weekend in empty classrooms and was 46-67%. It would have been better to measure the indoor climate during weekday, but the schools did not allow that. Due to the strong correlation between relative air humidity and type of school, we could not keep those two variables together in the regression models with mutual adjustment. However in the stepwise regression models, relative air humidity was retained in the models but the type of school was excluded. Higher relative air humidity in an empty classroom in the weekend may indicate a higher emission of dampness from the building or a lower ventilation flow. Higher relative air humidity in schools has been shown to be associated with higher levels of particles in the air (Norback et al., 1999) and higer relative air humidity in homes is associated with building dampness (Sahlberg et al., 2013).

Some limitations need to be considered in this study. Epidemiological studies may be affected by selection bias and information bias. The schools were arbitrarily selected in Hyogo, not based on any complaints on health and indoor problems. The participation rate was high (99.6%). Thus individual selection bias is less likely, but the study population may not be representative for all types of students in the area because of the limited number of schools. We investigated four schools, one public and three private and two of the private schools had only female students. This could have affected the representativeness of our study, but there were no differences in cat and dog allergen levels between private and public schools. Another weakness is the lack of clinical investigation. Recall bias is another potential problem in studies where data on exposure and medical symptoms is collected by same questionnaire, as for home factors in our study. In contrast, data on the school environment was collected by measurements, performed after questionnaire study was completed, and for school exposures recalled bias would not be a problem. Among home environment factor, we found consistent associations with SBS symptoms only for certain factors, such as signs of floor dampness and window pane condensation in winter. These factors are not well known as risk factors for SBS (or SHS) in Japan. On the contrary, potential sources of emissions of volatile organic compounds (VOC), such as new indoor painting or new floor materials, were not associated with SBS symptoms. This suggests that recall bias was not a major problem in our study. Another limitation is the cross-sectional design of the study which limits the possibility to draw conclusions on casually.

## 5. Conclusions

SBS symptoms were common among the Japanese junior high school students and were associated with both home and school environment factors. Window pane condensation and floor dampness at home can increase the risk for SBS symptoms. Moreover high relative air humidity or poor ventilation at school may increase the risk for SBS symptoms. This indicates a need to reduce dampness and increase the ventilation flow both in homes and schools in this area. Indoor home and school environmental exposure in Japan may have implications for health of students and can be an important public health issue.
